# Testing criteria for 22q11.2 deletion syndrome: preliminary results of a low cost strategy for public health

**DOI:** 10.1186/s13023-019-1098-1

**Published:** 2019-06-03

**Authors:** Ilária Cristina Sgardioli, Fabíola Paoli Monteiro, Paulo Fanti, Társis Paiva Vieira, Vera Lúcia Gil-da-Silva-Lopes

**Affiliations:** 1 Department of Medical Genetics and Genomic Medicine, Faculty of Medical Science, State University of Campinas (Unicamp), Tessália Vieira de Camargo Street, 126, Campinas, SP 13083-887 Brazil; 2 Association of Parents and Friends of the Exceptional from Sao Paulo (Associação de Pais e Amigos dos Excepcionais de São Paulo – APAE-SP), Campinas, SP Brazil; 3 Department of Statistics, Faculty of Medical Science, State University of Campinas (Unicamp), Campinas, SP Brazil

**Keywords:** 22q11.2 deletion syndrome, Clinical criteria, Diagnosis, Public health, Fluorescence in situ hybridization, Multiplex ligation probe-dependent amplification, Chromosomal microarray analysis

## Abstract

**Background:**

The clinical heterogeneity of the 22q11.2 Deletion Syndrome (22q11.2DS – OMIM, #188400 and #192430) is a universal challenge leading to diagnostic delay. The aim of this study was to evaluate a low cost strategy for the diagnosis of this condition based upon clinical criteria previously reported. Health professionals, who collected clinical data, from twelve centers were trained in those criteria, which were summed through an online application (*CranFlow*).

**Results:**

Clinical and laboratorial data of 347 individuals registered from 2008 to 2017 in the Brazilian Database on Craniofacial Anomalies/22q11.2 Deletion Syndrome, were reviewed. They were divided in two groups: (I) 168 individuals investigated before the definition of the criteria and (II) 179 individuals investigated after the criteria application. All of them were investigated for 22q11.2DS by Fluorescent in situ Hybridization (FISH) and/or Multiplex Ligation Probe-dependent Amplification (MLPA), detecting 98 cases with 22q11.2DS. Among the individuals with 22q11.2DS in Group II, 42/53 (79.25%) fulfilled the proposed criteria against 11/53 (20.75%) who did not fulfill them (*p* < .0001). The association of congenital heart diseases with high predictive value for 22q11.2DS and hypernasal voice were significantly associated to the presence of 22q11.2DS (*p* = 0.0172 and *p* < .0001, respectively). In addition, 22q11.2DS was confirmed 3.82 more times when the individuals fulfilled the proposed criteria. Of the 249 cases negative for the typical deletion in 22q11.2, Chromosomal Microarray Analysis (CMA) was performed in 132 individuals and detected pathogenic alterations at other genomic regions in 19 individuals, and variants of uncertain clinical significance in 31 cases.

**Conclusions:**

Therefore, a *locus-*specific approach could be used to individuals with positive criteria as a cost-effective alternative for 22q11.2DS diagnosis. The authors discuss advantages and suggest ways of implementing this approach to investigate 22q11.2DS in a public health system.

## Background

The 22q11.2 Deletion Syndrome (22q11.2DS – OMIM, #188400 and #192430) is the most common deletion in humans, with an estimated incidence of 1/4.000 to 1/5.000 births [1]. There is a wide range of clinical signs associated with this condition, being the most frequent: congenital heart disorders (mainly conotruncal defects), palatal anomalies, hypocalcemia, immunological deficiency, learning disability and developmental delay, behavioral and psychiatric disorders [2–7].

The diagnosis is based on the detection of the typical deletion in the 22q11.2 region, which comprises from 1.5 Mb to 3.0 Mb in size, without recognizable phenotypic differences between them [7, 8]. The evolution of laboratory methods in genetics has brought several diagnostic possibilities for this microdeletion, however, the wide clinical heterogeneity of this syndrome hampers the diagnosis.

In the mid-1990s, the Fluorescence in situ Hybridization (FISH) technique using a *locus*-specific probe for the 22q11.2 chromosome region was described, and for many years it has been considered as the “gold standard” for detecting this microdeletion [9–11]. This allows the detection of the proximal and common deletion in 22q11.2 and is the most widespread method. Nevertheless, a negative FISH result does not totally exclude the diagnosis of 22q11.2DS, since minor or atypical deletions may occur within the typically deleted region [12, 13]. In this context, technologies in molecular biology have allowed the development of different methods, such as the Multiplex Ligation-dependent Probe Amplification (MLPA) technique, an efficient, rapid and sensitive method for the diagnosis of 22q11.2DS, which detects both microdeletions and microduplications of this region, as well as alterations in other chromosomal regions that have been associated with the phenotype of this syndrome [10, 14]. More recently, methods for genome-wide analysis have achieved considerable visibility in the field of genetics, and currently, the chromosomal microarray analysis (CMA) is the first-tier technique for the investigation of the deletion in 22q11.2 [15]. Yet, its large-scale diffusion still fights the difficulties of high cost infrastructure and inputs in some countries.

The number of cases with the 22q11.2 deletion, among those with a clinical suspicion, is variable and dependent on the design of the study, varying from 4.0 to 78.2% [2, 8, 16–18]. Because of the clinical heterogeneity, even with different molecular techniques available, the time of diagnosis is still late, delaying the appropriate clinical management of the individual and genetic counseling of the family [2, 19, 20]. Outlining the clinical and/or phenotypic aspects that indicate 22q11.2 deletion screening and genotype-phenotype correlation studies have been a challenge and theme of interest to different researchers [9, 21–23].

In 2013, Monteiro and coworkers proposed clinical criteria to proceed with a laboratory investigation for 22q11.2DS, based upon a review of the literature and evaluation of 193 individuals. The main differential aspect of this proposal is the clinical general approach, which includes different manifestations of this microdeletion [22]. This article addresses the results of using these criteria in a group of 347 individuals with clinical suspicion of 22q11.2DS recorded in the Brazilian Database on Craniofacial Anomalies/22q11.2DS.

## Patients and methods

The cohort was composed of 347 individuals with clinical suspicion of 22q11.2DS or at least one major 22q11.2DS manifestation registered in the Brazilian Database on Craniofacial Anomalies/22q11.2 Deletion Syndrome (BDCA) from 2008 to 2017. Health professional participants of the Brazil’s CranioFacial Project (BCFP) evaluated all individuals. They were investigated for the 22q11.2DS by FISH with the *TUPLE1* probe (*Cytocell Aquarius*^®^ or *Visys Abbott*^©^) and/or MLPA with the P250 kit (*MRC-Holland*^®^, Amsterdam, the Netherlands).

The 347 individuals were evaluated for the proposed criteria in two periods. The first one included 168 individuals described by Monteiro et al. [22], to whom a standard clinical protocol including major features and some minor traits of 22q11.2DS had been applied (Group I). Individuals from Monteiro et al. [22] manuscript with schizophrenia as the major manifestation (Group IV of the original manuscript in which the criteria were proposed) were excluded since this study had a dysmorphologic approach. The second group was composed by individuals evaluated according to the clinical criteria proposed by the same authors, as described in Table 1 (Group II) [22].

**Table 1:** Clinical guidelines for screening the 22q11.2 deletion proposed by Monteiro et al. [22]

Column 1 – Absolute indications for confirmatory testing	Column 2 – Core features of the 22q11.2DS	Column 3 – Associated features of the 22q11.2DS
Any item from Column 1	At least two items from Column 2 OR one item from Column 2 and at least two items from Column 3	Two or more items from Column 3 and one from Column 2 OR at least four items from Column 3^a^
A. Cardiac malformation with high predictive value for the deletion: Interruption of aortic arch type B, Truncus arteriosus and/or Ventricular Septal defect with pulmonary atresia (Tetralogy of Fallot with pulmonary atresia) B. Neonatal hypocalcemia secondary to idiopathic hypoparathyreoidism	C. Other Conotruncal Heart defects: Classic Tetralogy of Fallot, Ventricular Septal Defect posterior malalignement, Ventricular Septal Defect Subarterial/Subpulmonary and/or Aortic Coarctation D. Palatal alterations: Velopharyngeal Insufficiency, Overt or submucous cleft palate and/or cleft lip/palate E. Immunodefficiency confirmed by labaratorial tests and/or thymic alterations (hypoplasia/aplasia) F. Typical Face with four or more characteristic dysmorphisms, being at least three among the following: Long face, hooded eyelids, tubular nose or other form of typical nose, alar hypoplasia G. Schizophrenia	H. Neurocognitive dysfunction: Neurodevelopmental delay, language development delay and/or learning disability I. Cardiovascular abnormalities: Aortic arch alterations and/or pulmonary arterial tree alterations J. Two or more suggestive dysmorphisms (> = 2 years-old) OR One or more suggestive dysmorphisms (<= 2 years-old) K. Hypernasal tone of voice L. Other cardiac defects: Other types of Ventricular Septal defect, Transposition of Great Arteries, Double right-outlet ventricle, Atrial septal defect and/or Patent ductus arteriosus M. Other Palatal alterations: Isolated bifid uvula and/or Cleft lip N. Genitourinary malformations

^a^ Patients younger than 1 year-old: One or more items from Column 3 and at least one item from Column 2 OR four or more items from Column 3 a Patients younger than 1 year-old: One or more items from Column 3 and at least one item from Column 2 OR four or more items from Column 3

Standardized clinical and complementary data were collected through a web-based application (*CranFlow*: Craniofacial anomalies: registration, flow, and management) [24]. It was developed by this research group for general craniofacial anomalies with a specific area for 22q11.2DS suspicion, in which data are recorded and the sum of features is automatically performed according to the proposed criteria by Monteiro [22]. Follow-up consultations are also recorded, allowing recognition of the individual’s and the diseases’ natural history, as well as updating the clinical manifestations and its sum, and differential diagnosis. This tool is available for interested researchers. Before data collection for Group II, all participant health professionals were trained to use the *CranFlow*.

Data analyses were performed using The Statistical Analysis System (SAS) for Windows, version 9.4 (SAS Institute INC, 2002–2008, Cary, NC, USA). Categorical variables were investigated by the Chi-Square test and Fischer’s exact test, when necessary. The logistic regression analysis was used to evaluate the factors that discriminate the 22q11.2DS among those proposed by Monteiro et al. [22]. The significance level was set at 5% (*p* < 0.05). The values of odds ratio (OR) and confidence interval (CI) were computed.

Additionally, 132 negative cases for 22q11.2DS had CMA through different platforms performed during clinical routine. All of these had CMA data reanalyzed in this study, following an “*in-house*” workflow based on standard recommendations [25].

## Results

The total sample included 347 individuals (186 females, 161 males) with clinical suspicion of 22q11.2 deletion syndrome. Group I was composed of 168 individuals (88 females, 80 males) and mean age at inclusion in BDCA was 11.6 years old. Group II included 179 individuals (98 females, 81 males) and the mean age at inclusion in BDCA was 7.9 years old. Overall, there was a difference of the mean age at inclusion among Groups I and II (*p* < .0001), indicating better access to a specialized service for group II. Among the individuals who were positive for 22q11.2DS in Groups I and II, the mean age at diagnosis was 12.7 against 8.8 years old (*p* = 0.0344), respectively. Table 2 shows the profile of the total sample comparing the proposed criteria and the laboratorial data in both groups.

**Table 2:** Description of the total sample, Groups I and II and positivity according to criteria proposed by Monteiro et.al [22]

	Positive 22q11.2DS	Negative 22q11.2DS	Total	*p* value^*^
Total sample	98/347 (28.24%)	249/347 (71.76%)	347 (100%)	<.0001
Positive for criteria	73/347 (21.04%)	122/347 (35.16%)	195/347 (56.20%)	–
Negative for criteria	25/347 (7.20%)	127/347 (36.60%)	152/347 (43.80%)	–
Group I^*^	45/168 (26.79%)	123/168 (73.21%)	168/347 (48.41%)	0.0628
Positive for criteria	31/96 (32.29%)	65/96 (67.71%)	96/168 (57.14%)	–
Negative for criteria	14/72 (19.44%)	58/72 (80.56%)	72/168 (42.86%)	–
Group II - Total	53/179 (29.61%)	126/179 (70.39%)	179/347 (51.59%)	<.0001
Positive for criteria	42/99 (42.42%)	57/99 (57.58%)	99/179 (55.31%)	–
Negative for criteria	11/80 (13.75%)	69/80 (86.25%)	80/179 (44.69%)	–

^*^p value according to main groups

Comparing individuals with 22q11.2DS in the Groups I and II, the percentage at diagnosis of the 22q11.2DS was 26.79% against 29.61%, respectively (*p* = 0.5593). However, in Group II, the presence of 22q11.2 deletion in individuals with positive criteria (42/99–42.42%) was significant when compared to the number of individuals negative for the criteria (11/80–13.75%) (*p* < .0001). The same comparison in Group I, did not show a significant difference, 31/96 (32.29%) against 14/72 (19.44%), respectively (*p* = 0.0628).

Among the 99 individuals with positive criteria in Group II, the general distribution according to different criteria groups proposed by Monteiro et al. [22] were 10 for column 01 (10.10%), 24 for column 02 (24.24%), 6 for column 03 (6.06%) and 59 for the combination between columns 02 and 03 (59.60%) (*p* = 0.0004). Moreover, the association of a congenital heart disease with high predictive value for 22q11.2DS (items A) and hypernasal tone of voice (item K) was strongly indicative of 22q11.2DS in Group II (*p* = 0.0172 and *p* < .0001, respectively).

Among the confirmed cases for 22q11.2DS in the Group II, the analysis of the proposed criteria, positive (42/99–42.42%) or negative (11/80–13.75%), revealed that the criteria were significant (*p* < .0001) and represents an increased diagnosis of 3.82 times. Table 3 shows the results of the analysis by the items of criteria.

**Table 3:** Comparison among different items from the criteria proposed by Monteiro et al. [22] in Group II

Items of criteria according to Table 1	Positive criteria	Total of Individuals	Negative for 22q11.2DS	Positive for 22q11.2DS	*p* value	OR^a^	IC95%^b^
A	Yes	09	04 (3.47%)	05 (9.43%)	0.0949	3.177	0.818–12.336
	No	170	122 (96.83%)	48 (90.57%)	–	1.000	–
B#	Yes	–	–	–	–	–	–
	No	–	–	–	–	–	–
C	Yes	22	15 (11.90%)	07 (13.21%)	0.9086	1.126	0.431–2.943
	No	157	111 (88.1%)	46 (86.79%)	–	1.000	–
D	Yes	101	65 (51.59%)	36 (67.92%)	0.0459	1.987	1.013–3.901
	No	78	61 (48.41%)	17 (32.08%)	–	1.000	–
E	Yes	11	08 (6.35%)	03 (5.66%)	0.8610	0.885	0.225–3.474
	No	168	118 (93.65%)	50 (94.34%)	–	1.000	–
F	Yes	22	13 (10.32%)	09 (16.98%)	0.2193	1.778	0.710–4.455
	No	157	113 (89.68%)	44 (83.02%)	–	1.000	–
G##	Yes	–	–	–	–	–	–
	No	–	–	–	–	–	–
H	Yes	76	48 (38.10%)	28 (52.83%)	0.0702	1.820	0.952–3.480
	No	103	78 (61.90%)	25 (47.17%)	–	1.000	–
I	Yes	03	02 (1.59%)	01 (1.89%)	0.8868	1.192	0.106–13.438
	No	176	124 (98.41%)	52 (98.11%)	–	1.000	–
J	Yes	94	61 (48.41%)	33 (62.26%)	0.0919	1.758	0.912–3.388
	No	85	65 (51.59%)	20 (37.74%)	–	1.000	–
K	Yes	66	34 (26.98%)	32 (60.38%)	<.0001	4.123	2.096–8.111
	No	113	92 (73.02%)	21 (39.62%)	–	1.000	–
L	Yes	66	44 (34.92%)	22 (41.51%)	0.4048	1.323	0.685–2.553
	No	113	82 (65.08%)	31 (58.49%)	–	1.000	–
M	Yes	08	07 (5.56%)	01 (1.89%)	0.3015	0.327	0.028–2.725
	No	171	119 (94.44%)	52 (98.11%)	–	1.000	–
N	Yes	11	10 (7.94%)	1 (1.89%)	0.1578	0.223	0.028–1.788
	No	168	116 (92.06%)	52 (98.11%)	–	1.000	–

The criteria groups are described according to the criteria proposed by Monteiro et.al. [22] in Table 1

^a^OR - odds ratio for deletion

^b^IC95% - ratio confidence interval. B #: there were not enough cases for analysis; G ##: individuals with schizophrenia were not evaluated in this study

Of the 249 negative cases for 22q11.2DS, 117 are still in follow up and 132 were investigated through CMA, which detected chromosomal imbalances at other genomic regions in 19 individuals, including five atypical deletions in 22q11.2. Besides, variants of uncertain clinical significance (VUS) were detected in 31 cases, which require other laboratory techniques for conclusion, such as three cases suggestive of Uniparental Disomy (UDP) which are still under investigation. CMA was normal 82 cases.

## Discussion

### Testing the clinical criteria for 22q11.2DS

Phenotypic heterogeneity of the 22q11.2DS is known to pose difficulties to clinical suspicion of this condition, especially in cases with mild manifestations. Most previous studies on this subject focused on groups of individuals with similar clinical signs. This approach does not allow reaching a general consensus for the investigation of 22q11.2DS, leading to difficulties in clinical practice [2, 7, 19, 26, 27].

An important aspect of the clinical criteria proposed by Monteiro et al. [22] is the inclusion of distinct manifestations of 22q11.2DS, as well as different combinations of those clinical signs, allowing a wider approach to patient selection for testing and phenotypic recording [22]. However, its applicability has never been tested. Therefore, this is the first study using these criteria.

In addition, this study has another differential that can aggregate in clinical practice and in public health, which is the introduction of an online tool - *CranFlow*, which standardizes the data collection according to those criteria, as well as facilitates their summation by groups and columns for each consultation, since it is performed automatically in each evaluation [24].

The analysis of the total sample revealed the presence of 22q11.2DS in 98 individuals, of whom 73 (74.49%) fulfilled the proposed criteria and 25 (25.51%) did not. According to these results, the proposed criteria would contribute to the diagnostic conclusion of 2/3 of 22q11.2DS cases. Differences among Groups I and II probably were related to the inclusion criteria, as well as to the strategy of data collection. Group I was composed of individuals investigated according to clinical suspicion of 22q11.2DS by a medical geneticist, or by the presence of at least one major manifestation of this condition. Moreover, clinical data recorded included mainly more characteristic manifestations of the 22q11.2DS. It is therefore possible that some individuals from Group I had additional minor features of this disorder that were not reported by the health care provider. Conversely, Group II was designed including training for recognition and recording of a wide spectrum of 22q11.2DS features based upon the proposed criteria.

Therefore, the results from Group II are more reliable when it comes to testing the proposed criteria. They demonstrate a significant difference in patients who tested positive for 22q11.2DS and fulfill the proposed criteria, being a large proportion of those individuals with a combination of columns 02 and 03, i.e., patients with one core manifestation plus associated (although more unspecific) features. Overall, the criteria increased by 3.82 times the diagnosis when comparing individuals with confirmed 22q11.2DS positive or negative for the proposed criteria, suggesting its applicability on large scale.

Despite sample size, which did not allow to accurately identify statistical significance for each individual item from the three columns, it was possible to determine that the presence of a congenital heart disease with high predictive value for 22q11.2DS (item A) and hypernasal tone of voice (item K) was strongly indicative of 22q11.2DS in Group II (*p* = 0.0172 and *p* < .0001, respectively), suggesting consistency of the proposed criteria.

The criteria proposed by Monteiro et al. [22] show an alternative for reducing the age at diagnosis, which is a current and universal theme. The mean age at diagnosis of 22q11.2DS in two Brazilian studies were around 10 years old [2, 20], which is similar to studies of other countries according to Palmer and coworkers [19].

Mean age at inclusion in the database among Groups I and II was significantly decreased, but still did not affected the mean age at 22q11.2DS diagnosis. This could be explained by difficulties in access to genetic clinical evaluation and laboratory investigation in Brazil [28–33]. Even so, the sample was not large enough to verify the repercussion on age at diagnosis of 22q11.2DS.

Considering the duration of the study, the prevalence of 22q11.2DS and the comprehensiveness of the criteria, the results described here seem quite promising. The extended application in different populations could potentially bring more insight into the current research strategy. In the long term, it also could contribute to the refinement or modification of the criteria herein tested.

### Diagnosis of 22q11.2DS and public health

The design of this study is consistent and allows interpretations and immediate proposals for health care. A highlight is the laboratory approach that may be interesting for public health application, especially in countries where access to technology, considering infrastructure and inputs, is limited due to high costs.

Considering that 73 out of the 98 patients who tested positive for the typical deletion fulfilled the proposed criteria, a hierarchical laboratory approach could be applied in which, in the presence of proposed clinical criteria, *locus*-specific tests, such as FISH and MLPA, would be primary used. Despite international recommendations to use CMA as first-tier test for individuals with intellectual disability (ID) and/or multiple congenital anomalies (MCA) [34], the approach based on training for phenotypic recognition and use of a *locus*-specific test would allow for earlier diagnosis of a greater number of individuals with less financial impact. It would be of particular interest in regions in which expenditures with healthcare are scarce.

As an example, an estimated comparison of costs for CMA and *locus*-specific tests was described. These costs were based on the values indicated and published by the National Committee for the Implantation of Technology (Comitê Nacional de Implantação de Tecnologia - CONITEC) from the Unified Health Care System (Sistema Único de Saúde - SUS) describing laboratory procedures for the diagnosis of rare diseases associated with congenital anomalies. The converted values are equivalent to R$ 3.70 (Brazilian currency - Real - R$).

Currently, the CMA is the first-tier screening test indicated for the investigation of genomic imbalances, in individuals with multiple congenital anomalies and (or) developmental delay / intellectual deficiency. The value proposed by CONITEC for CMA test is U$ 540.54, which represents U$ 540,540.54 for the investigation of 1000 individuals. The values estimated in the same document for the FISH and MLPA methods present the cost of U$ 55.39 and U$ 52.14 per test respectively. Adopting the cost of U$ 54.05 for *locus*-specific test, the investigation of 1000 individuals would have an initial cost of U$ 54,054.05. Applying the 42.42%, of positivity rate of the individuals who fulfilled the criteria of Group II in the present study, complementary investigation by CMA for the other 576 individuals without 22q.11.2DS would add U$ 311,351.35. Therefore, the final cost using the proposed approach would be U$ 365,405.41 against U$ 540,540.54 with CMA as the first-tier diagnostic test. It represents a saving of U$ 175,135.13, which allows a significant economic impact.

Jehee et al. [35] also showed that the application of *locus*-specific tests as first-tier tests proved to be quite effective and is an important alternative for diagnostic screening in countries where health care costs are scarce. In 2014, Geddes and colleagues carried out a cost-effectiveness survey and proposed that each institution/system should evaluate the results of its genetic tests, within its possibilities, to develop protocols of high yield within a cost-effective standard [36].

In this context, applying the proposed criteria combined with initial screening by *locus*-specific tests, such as FISH or MLPA, can prove to be an important strategy for the initial diagnostic investigation of 22q11.2DS in countries where major financial constraints are an issue or even to optimize financial resources in health care.

The present study allowed the diagnosis of 98 individuals with 22q11.2DS and 19 more individuals with genomic imbalances in other regions by CMA. In long time, recording this data in *CranFlow* would allow recognize disorders which overlap with 22q11.2DS. In addition, the reanalysis of CMA in this study showed the importance of the standardization of CMA data analysis, as has been demonstrated by other authors [25, 37]. All these data shows important points for discussion for public health regarding 22q11.2DS and other chromosomal disorders detected by CMA.

It is noteworthy that, all the diagnostic methods described in this work have advantages and disadvantages, being necessary the evaluation of their applicability, as well as the local infrastructure resources must be considered. CMA, although requiring more technological equipment, allows conclusion in around 15 to 20% of cases [34]. In this study, CMA resulted in increased diagnostic rate, which is in accordance to the report by Koczkowska and coworkers that described an increase in diagnostic yield by approximately 12% [23]. Additionally, CMA detects cases in which other molecular and cytogenetic techniques will be needed for definite conclusion, thus demanding a specialized laboratory.

Considering the current options for 22q11.2 deletion testing (*locus-*specific or genome wide methods), individuals meeting the proposed criteria could be offered a *locus-*specific approach, whereas individuals not fulfilling them would probably benefit from a genome wide method, such as CMA. In view of the global discrepancies in access for genetic tests, this strategy could be a cost-effective choice for 22q11.2DS diagnosis.

The data obtained evidence the difficulties in 22q11.2DS diagnosis and reinforces the phenotypic heterogeneity of this condition, which clinically overlaps with other genomic alterations. The proposed approach with hierarchical use of tests and centralization of more elaborate laboratory techniques, as well as standardization of unified pattern of analysis and interpretation of data, is a relevant strategy for diagnosis in any public health system.

## Conclusion and final suggestions

Considering the proposed strategy for 22q11.2DS diagnosis in public health care, the following suggestions could be adopted: a) training of health care provider teams; b) applying proposed clinical criteria and using *CranFlow* online tool to record clinical data and follow up patients with 22q11.2DS suspicion; c) hierarchy of genetic tests where CMA is not readily available as the first-tier technique; and d) centralization for specialized techniques; e) standardizing the analysis and interpretation of genetic data and f) creation of a national registry to optimize research resources in planning public health policies for the diagnosis of 22q11.2DS.

These suggestions may bring important evidences for public health for 22q11.2DS diagnosis, such as recognition and importance of rare features, as well as the differential diagnosis of this condition, and the economic impact of the approach herein proposed.

## Data Availability

Not applicable.

## Abbreviations

22q11.2DS: 22q11.2 Deletion Syndrome
APAE: Association of Parents and Friends of the Exceptional from Sao Paulo (Associação de Pais e Amigos dos Excepcionais de São Paulo – APAE-SP)
BCFP: Brazil’s CranioFacial Project
BDCA: Brazilian Database on Craniofacial Anomalies/22q11.2 Deletion Syndrome
CI: Confidence Interval
CMA: Chromosomal Microarray Analysis
CONITEC: National Committee for the Implantation of Technology (Comitê Nacional de Implantação de Tecnologia)
*CranFlow*: Craniofacial Anomalies: registration, flow, and management
FISH: Fluorescent in situ Hybridization
ID: Intellectual Disability
MCA: Multiple Congenital Anomalies
MLPA: Multiplex Ligation Probe-dependent Amplification
OMIM: Online Mendelian Inheritance in Man
OR: Odds Ratio
SAS: Statistical Analysis System
SUS: Unified Health Care System (Sistema Único de Saúde - SUS)
UDP: Uniparental Disomy
VUS: Variants of Uncertain Clinical Significance

## References

[1] Swillen A, Vogels A, Devriendt K, Fryns JP (2000). Chromosome 22q11 deletion syndrome: update and review of the clinical features, cognitive-behavioral spectrum, and psychiatric complications. Am J Med Genet.

[2] Vieira TP, Monteiro FP, Sgardioli IC, Souza J, Fett-Conte AC, Monlleó IL, Fontes MB, Félix TM, Leal GF, Ribeiro EM, Gil-da-Silva-Lopes VL (2015). Clinical features in patients with 22q11.2 deletion syndrome ascertained by palatal abnormalities. Cleft Palate Craniofac J.

[3] Crowley B, Ruffner M, McDonald McGinn DM, Sullivan KE (2018). Variable immune deficiency related to deletion size in chromosome 22q11.2 deletion syndrome. Am J Med Genet A.

[4] Unolt M, Versacci P, Anaclerio S, Lambiase C, Calcagni G, Trezzi M, Carotti A, Crowley TB, Zackai EH, Goldmuntz E (2018). Congenital heart diseases and cardiovascular abnormalities in 22q11.2 deletion syndrome: from well-established knowledge to new frontiers. Am J Med Genet A.

[5] Rayannavar A, Levitt Katz LE, Crowley TB, Lessig M, Grand K, Goldmuntz E, Zackai EH, McDonald-McGinn DM (2018). Association of hypocalcemia with congenital heart disease in 22q11.2 deletion syndrome. Am J Med Genet A.

[6] Swillen A, Moss E, Duijff S (2018). Neurodevelopmental outcome in 22q11.2 deletion syndrome and management. Am J Med Genet A.

[7] McDonald-McGinn DM, Sullivan KE, Marino B, Philip N, Swillen A, Vorstman JA, Zackai EH, Emanuel BS, Vermeesch JR, Morrow BE (2015). 22q11.2 deletion syndrome. Nat Rev Dis Primers.

[8] Wu D, Chen Y, Xu C, Wang K, Wang H, Zheng F, Ma D, Wang G (2013). Characteristic face: a key indicator for direct diagnosis of 22q11.2 deletions in Chinese velocardiofacial syndrome patients. PLoS One.

[9] Oh AK, Workman LA, Wong GB (2007). Clinical correlation of chromosome 22q11.2 fluorescent in situ hybridization analysis and velocardiofacial syndrome. Cleft Palate Craniofac J.

[10] Fernandez L, Lapunzina P, Arjona D, Lopez Pajares I, Garcia-Guereta L, Elorza D, Burgueros M, De Torres ML, Mori MA, Palomares M (2005). Comparative study of three diagnostic approaches (FISH, STRs and MLPA) in 30 patients with 22q11.2 deletion syndrome. Clin Genet.

[11] Sgardioli IC, Vieira TP, Simioni M, Monteiro FP, Gil-da-Silva-Lopes VL (2015). 22q11.2 deletion syndrome: laboratory diagnosis and TBX1 and FGF8 mutation screening. J Pediatr Genet.

[12] Driscoll DA, Salvin J, Sellinger B, Budarf ML, McDonald-McGinn DM, Zackai EH, Emanuel BS (1993). Prevalence of 22q11 microdeletions in DiGeorge and velocardiofacial syndromes: implications for genetic counselling and prenatal diagnosis. J Med Genet.

[13] Poirsier C, Besseau-Ayasse J, Schluth-Bolard C, Toutain J, Missirian C, Le Caignec C, Bazin A, de Blois MC, Kuentz P, Catty M, et al. A French multicenter study of over 700 patients with 22q11 deletions diagnosed using FISH or aCGH. Eur J Hum Genet. 2015. 10.1038/ejhg.2015.219 PMC4867458 26508576

[14] Jalali GR, Vorstman JA, Errami A, Vijzelaar R, Biegel J, Shaikh T, Emanuel BS (2008). Detailed analysis of 22q11.2 with a high density MLPA probe set. Hum Mutat.

[15] McDonald-McGinn DM, Sullivan KE (2011). Chromosome 22q11.2 deletion syndrome (DiGeorge syndrome/velocardiofacial syndrome). Medicine (Baltimore).

[16] Peyvandi S, Lupo PJ, Garbarini J, Woyciechowski S, Edman S, Emanuel BS, Mitchell LE, Goldmuntz E (2013). 22q11.2 deletions in patients with conotruncal defects: data from 1,610 consecutive cases. Pediatr Cardiol.

[17] Cuturilo G, Drakulic D, Jovanovic I, Krstic A, Djukic M, Skoric D, Mijovic M, Stefanovic I, Milivojevic M, Stevanovic M (2016). Improving the diagnosis of children with 22q11.2 deletion syndrome: a single-center experience from Serbia. Indian Pediatr.

[18] Geddes GC, Basel D, Frommelt P, Kinney A, Earing M (2017). Genetic testing protocol reduces costs and increases rate of genetic diagnosis in infants with congenital heart disease. Pediatr Cardiol.

[19] Palmer LD, Butcher NJ, Boot E, Hodgkinson KA, Heung T, Chow EWC, Guna A, Crowley TB, Zackai E, McDonald-McGinn DM, Bassett AS (2018). Elucidating the diagnostic odyssey of 22q11.2 deletion syndrome. Am J Med Genet A.

[20] Grassi MS, Jacob CM, Kulikowski LD, Pastorino AC, Dutra RL, Miura N, Jatene MB, Pegler SP, Kim CA, Carneiro-Sampaio M (2014). Congenital heart disease as a warning sign for the diagnosis of the 22q11.2 deletion. Arq Bras Cardiol.

[21] Kruszka P, Addissie YA, McGinn DE, Porras AR, Biggs E, Share M, Crowley TB, Chung BH, Mok GT, Mak CC (2017). 22q11.2 deletion syndrome in diverse populations. Am J Med Genet A.

[22] Monteiro FP, Vieira TP, Sgardioli IC, Molck MC, Damiano AP, Souza J, Monlleó IL, Fontes MI, Fett-Conte AC, Félix TM (2013). Defining new guidelines for screening the 22q11.2 deletion based on a clinical and dysmorphologic evaluation of 194 individuals and review of the literature. Eur J Pediatr.

[23] Koczkowska M, Wierzba J, Smigiel R, Sasiadek M, Cabala M, Slezak R, Iliszko M, Kardas I, Limon J, Lipska-Zietkiewicz BS (2017). Genomic findings in patients with clinical suspicion of 22q11.2 deletion syndrome. J Appl Genet.

[24] Volpe-Aquino RM, Monlleó IL, Lustosa-Mendes E, Mora AF, Fett-Conte AC, Félix TM, Xavier AC, Tonocchi R, Ribeiro EM, Pereira R (2018). CranFlow: an application for record-taking and management through the Brazilian database on craniofacial anomalies. Birth Defects Res.

[25] de Souza LC. Phenotype comparison among individuals with developmental delay / intellectual disability with or without genomic imbalances. In: dos Santos AP, Sgardioli IC, Viguetti-Campos NL, JRM P, de Oliveira-Sobrinho RP, Vieira TP, Gil-da-Silva-Lopes VL, editors. Phenotype comparison among individuals with developmental delay / intellectual disability with or without genomic imbalances: J Intellect Disabil Res; 2018. 10.1111/jir.12615 30900361

[26] Hay BN (2007). Deletion 22q11: spectrum of associated disorders. Semin Pediatr Neurol.

[27] Fernandez L, Nevado J, Santos F, Heine-Suner D, Martinez-Glez V, Garcia-Minaur S, Palomo R, Delicado A, Pajares IL, Palomares M (2009). A deletion and a duplication in distal 22q11.2 deletion syndrome region. Clinical implications and review. BMC Med Genet.

[28] Marques-de-Faria AP, Ferraz VE, Acosta AX, Brunoni D (2004). Clinical genetics in developing countries: the case of Brazil. Community Genet.

[29] Horovitz DD, Cardoso MH, Llerena JC, de Mattos RA (2006). Birth defects in Brazil and health care: proposals for public policies in clinical genetics. Cad Saude Publica.

[30] Monlleó IL, Gil-da-Silva-Lopes VL (2006). Brazil's craniofacial project: genetic evaluation and counseling in the reference network for craniofacial treatment. Cleft Palate Craniofac J.

[31] Castilla EE, Luquetti DV (2009). Brazil: public health genomics. Public Health Genomics.

[32] Monlleo IL, Mossey PA, Gil-da-Silva-Lopes VL (2009). Evaluation of craniofacial care outside the Brazilian reference network for craniofacial treatment. Cleft Palate Craniofac J.

[33] Vieira TP, Sgardioli IC, Gil-da-Silva-Lopes VL (2013). Genetics and public health: the experience of a reference center for diagnosis of 22q11.2 deletion in Brazil and suggestions for implementing genetic testing. J Community Genet.

[34] Miller DT, Adam MP, Aradhya S, Biesecker LG, Brothman AR, Carter NP, Church DM, Crolla JA, Eichler EE, Epstein CJ (2010). Consensus statement: chromosomal microarray is a first-tier clinical diagnostic test for individuals with developmental disabilities or congenital anomalies. Am J Hum Genet.

[35] Jehee FS, Takamori JT, Medeiros PF, Pordeus AC, Latini FR, Bertola DR, Kim CA, Passos-Bueno MR (2011). Using a combination of MLPA kits to detect chromosomal imbalances in patients with multiple congenital anomalies and mental retardation is a valuable choice for developing countries. Eur J Med Genet.

[36] Geddes GC, Butterly M, Sajan I (2015). FISH for 22q11.2 deletion not cost-effective for infants with congenital heart disease with microarray. Pediatr Cardiol.

[37] Palmer E, Speirs H, Taylor PJ, Mullan G, Turner G, Einfeld S, Tonge B, Mowat D (2014). Changing interpretation of chromosomal microarray over time in a community cohort with intellectual disability. Am J Med Genet A.

